# Case Report: Two Monochorionic Twins With a Critically Different Course of Progressive Osseous Heteroplasia

**DOI:** 10.3389/fped.2021.662669

**Published:** 2021-06-23

**Authors:** Antonio José Justicia-Grande, Jose Gómez-Ríal, Irene Rivero-Calle, Sara Pischedda, María José Curras-Tuala, Alberto Gómez-Carballa, Miriam Cebey-López, Jacobo Pardo-Seco, Roberto Méndez-Gallart, María José Fernández-Seara, Antonio Salas, Federico Martinón-Torres

**Affiliations:** ^1^Genetics, Vaccines, Infectious Diseases and Pediatrics Research Group (GENVIP Group), Instituto de Investigación Sanitaria de Santiago de Compostela, Santiago de Compostela, Spain; ^2^Physical Medicine and Rehabilitation Department, Hospital Clínico Universitario de Santiago de Compostela, A Coruña, Spain; ^3^Immunology Laboratory, Clinical Laboratory, Hospital Clínico Universitario Santiago de Compostela, A Coruña, Spain; ^4^Translational Pediatrics and Infectious Diseases, Department of Pediatrics, Hospital Clínico Universitario de Santiago de Compostela, A Coruña, Spain; ^5^Pediatric Surgery, Hospital Clínico Universitario de Santiago de Compostela, A Coruña, Spain; ^6^Unidade de Xenética, Instituto de Ciencias Forenses, Facultade de Medicina, Universidade de Santiago de Compostela, Santiago de Compostela, Spain; ^7^GenPoB Research Group, Instituto de Investigaciones Sanitarias, Hospital Clínico Universitario de Santiago de Compostela, A Coruña, Spain

**Keywords:** progressive osseous heteroplasia, POH, treatment, genetic diseases, monochorionic twins

## Abstract

Progressive osseous heteroplasia (POH; OMIM 166350) is a rare autosomal-dominant genetic disorder in which extra-skeletal bone forms within skin and muscle tissue. POH is one of the clinical manifestations of an inactivating mutation in the *GNAS* gene. *GNAS* gene alterations are difficult matter to address, as *GNAS* alleles show genetic imprinting and produce several transcript products, and the same mutation may lead to strikingly different phenotypes. Also, most of the publications concerning POH patients are either clinical depictions of a case (or a case series), descriptions of their genetic background, or a tentative correlation of both clinical and molecular findings. Treatment for POH is rarely addressed, and POH still lacks therapeutic options. We describe a unique case of POH in two monochorionic twins, who presented an almost asymptomatic vs. the severe clinical course, despite sharing the same mutation and genetic background. We also report the results of the therapeutic interventions currently available for heterotopic ossification in the patient with the severe course. This article not only critically supports the assumption that the POH course is strongly influenced by factors beyond genetic background but also remarks the lack of options for patients suffering an orphan disease, even after testing drugs with promising *in vitro* results.

## Introduction

Progressive osseous heteroplasia (POH; OMIM 166350) is a rare autosomal-dominant genetic disorder in which extra-skeletal bone forms within skin and muscle tissue (1). Most cases of POH are caused by heterozygous inactivating mutations of the *GNAS* gene on the paternal allele (2, 3). *GNAS* encodes the alpha subunit of the G-stimulatory protein of adenylyl cyclase (Gsα). The scarce cases, the variability in clinical presentation, and the unpredictable evolution hamper the study of the disease, which in many cases leads to deformities, ankylosis, loss of functionality, and even amputation (4, 5).

We describe for the first time the case of two monochorionic twins suffering from POH, with almost asymptomatic vs. severe clinical course of the disease. Our aim is 2-fold: (i) to bring the focus on the different clinical evolution despite patients having an identical genetic background and (ii) to review the treatment options for severe POH and communicate our experience.

## Case Presentation

Two 4-year-old monochorionic–diamniotic twins with a diagnosis of POH were referred to our practice for evaluation and follow-up. Clinical evolution and analytical findings of both siblings can be seen in Table 1 and Supplementary Figure 1. Their condition as monozygotic twins was tested by carrying out whole-genome single nucleotide polymorphism (SNP) genotyping (including 715.195 SNPs) of blood and saliva samples obtained from both sisters and a sample of bone plate from the severe affected twin. The statistical analyses confirmed that the twins are genetically identical and also the absence of detectable mosaicism in the tissue samples analyzed (Supplementary Figure 2).

**Table 1:** Clinical history of both patients.

**Variables**	**Patient 1**	**Patient 2**
Birth	Pre-term—31 weeks of gestational age	
	No deformities at birth	
Family history	Mother: vitiligo, hypothyroidism, migraine	
	Father: bilateral calcifications after tearing of both Achilles' tendons	
	No Intermarriage between parental families	
Initial symptoms	10 weeks of extrauterine life	
	Hard subcutaneous papules in both tights	Three minimal subcutaneous spikes that remained stationary to date
Genetic characterization	Heterozygous missense mutation in exon 7 consisting of a 4 bp deletion (GACT; GNAS n565-568; 20q13), *de novo* mutation	
Progression of the disease	Progressed rapidly: Ankylosis of the left leg and calcification of deep tissues in the right leg, her back and the adipose tissue of both iliac fossae. At the time of their first visit to our practice, the illness started to impair joints of the right lower limb (the one still allowing walking—Supplementary Figure 1) Ibuprofen (8 mg/kg/dose) was required to mitigate pain	Remained largely unaffected by the disease. No new calcifications.
**Biochemical and hematological parameters**		
Insulin Growth Factor-1 (ng/mL).	39.7	56.2
NR: 49-327 ng/mL		
Growth hormone (ng/mL). NR: <5.00	1.28	1.07
Bone Alkaline Phosphatase (BAP) levels (mcg/L). NR: 41-134	236	122
Amino-terminal propeptide of type I collagen (PINP) (ng/mL). NR: 277-824	853.6	666.7
Beta carboxy-terminal telopeptide of type 1 collagen (beta-crosslaps) (ng/mL). NR: 0.57-1.84	1.71	1.29
Serum calcium (mg/dL). NR: 9.2-10.3	10.2	10.3
25hydroxy-vitamin D (ng/mL)	25	21
NR: 12-54		
Ionic phosphate (mg/dL). NR: 3.5-5.5	3.9	5
Osteocalcin (ng/mL)	47.2	25.4
NR: 2.8-41		
Parathyroid hormone (pg/mL)	35	36
NR: 9-59		
Tyroid stimulating hormone (TSH) (mIU/L). NR: 0.35-5.50	1.92	2.52
**Autoantibodies**		
ANA*	Negative	Negative
AMA*	Negative	Negative
ANCA*	Negative	Negative
RF* (NR 35-60UI/dL)	<35	<35
A-Sm*	Negative	Negative

*The girls are monochorionic-diamniotic twins and therefore share the same genetic background.*

ANA*, anti-nuclear antibodies; AMA*, anti-mitochondrial antibodies; ANCA*, anti-neutrophil cytoplasmic antibodies; RF*, rheumatoid factor; A-Sm*, anti-Smith antibody; NR, normal range.

**Figure 1:**
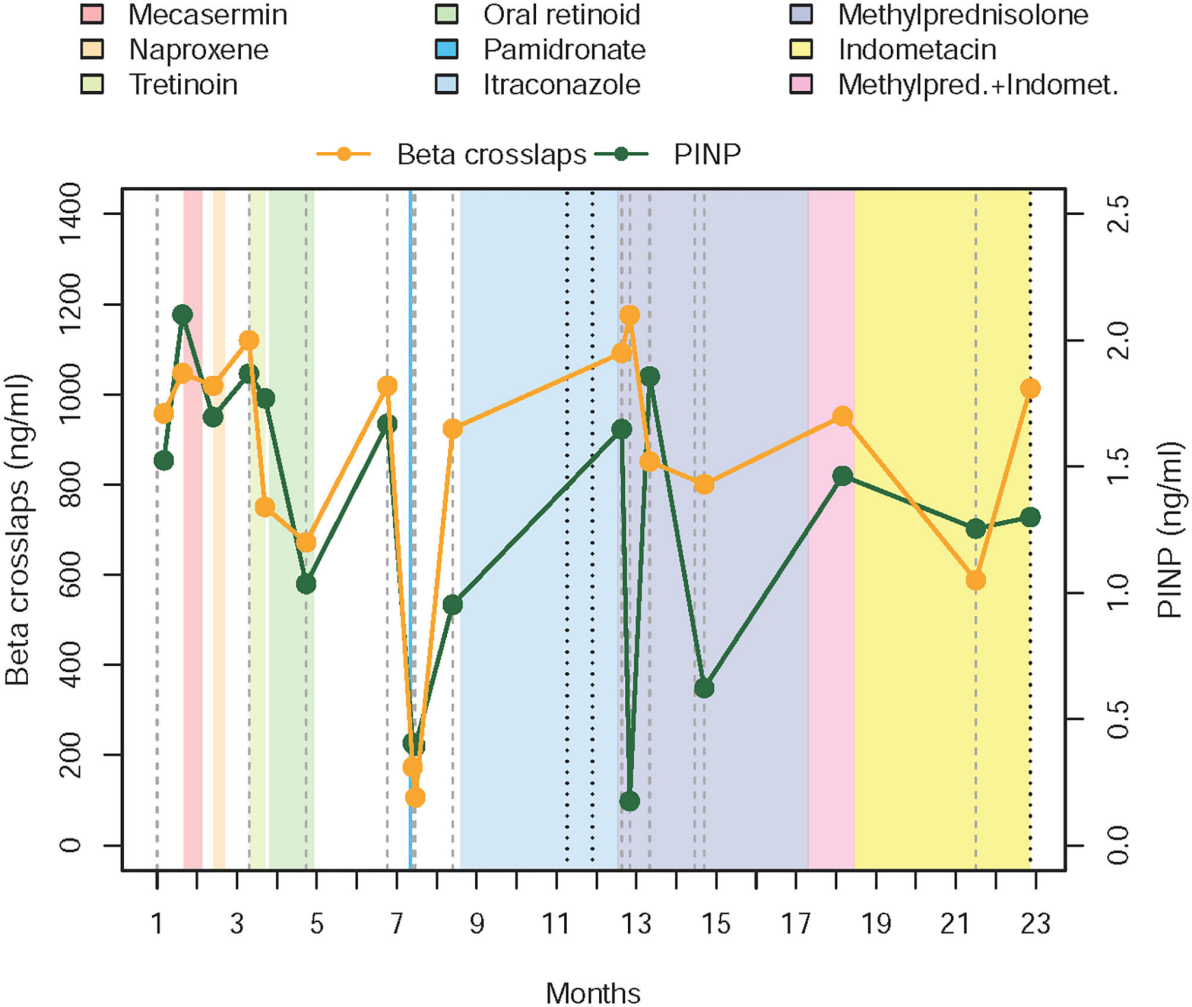
Chronogram depicting treatments received and evolution of the disease in the first twin. The graphic starts with levels of PINP and beta-crosslaps obtained in the 4 weeks prior to the beginning of the first intervention (administration of mecasermin or rhIGF-1, which corresponds to the beginning of month II). Levels of PINP and beta-crosslaps have been moving around the same values in the previous 12 months, where we began to measure them. After the failure of the corticosteroid therapy, parents and the medical team agreed to stop the fortnightly blood draws and to follow evolution based on clinical parameters, to reduce intervention and hospital visits, thus minimizing the interference with daily activities. PINP—amino-terminal propeptide of type I collagen.

Due to the rapid progression of disease and risk of permanent disability in the first twin, a review of the therapeutic options recorded in the literature was carried out and a subsequent treatment protocol was devised. The order of the therapeutic choices was established weighing the existing evidence for treatment of ectopic ossification processes against the severity of possible side effects and the disruption caused in normal life activities (Figure 1; Supplementary Figure 3). As the condition of the second twin remained stationary, interventions were only applied to the first sibling. All treatments included were approved as compassionate drug use by all the departments involved (Pediatrics, Neonatology, Endocrinology, Gastroenterology, Immunology and Infectious Diseases, Neurology, Pediatric Surgery, Dermatology, Chronic Patients Unit, Traumatology, Rehabilitation and Physiotherapy, Pharmacy, and Laboratory).

We chose to assess procollagen type I N-terminal peptide (PINP) and beta-cross lap levels during protocol follow-up. For that purpose, blood samples were drawn before starting a new treatment, fortnightly while maintaining that therapy and after drug withdrawal. Flare-ups were defined as periods of asthenia and muscular complaint that extended for five or more days, accompanied by evidence of progression of already existent lesions or formation of new ones.

A chronogram depicting the drugs administered to Patient 1 and their effects on the chosen serum markers can be found in Figure 1. Table 2 resumes dosing, adverse events found, and causes for discontinuation of the selected therapies.

**Table 2:** Drugs used in Patient 1.

**Drug**	**Dose**	**Mechanism**	**AL**	**Adverse events**	**Cause of discontinuation**	**References**
Mecasermin (rhIGF-1)	0.04 mg/day	rhIGF-I	15	No	Worsened serum markers; same clinical	(6–8)
Naproxen	100 mg	NSAIDs	40	Aphtous ulcers	Aphtous ulcers	(9)
Topical Tretinoin	0.10% 0.025%	Retinoid: Stimulation of Gsα expression at a transcriptional level	10	Red, swollen rash in the chosen regions	Ossification over the scapula grew	(10–12)
Oral acitretin	10 mg/day		90	No	Coalescence of bony spikes of the back and progression of the plate over the left scapula, as well as appearance of new spikes surrounding the abdominal plates.	(13)
Pamidronate	2.5 mg/kg	Bisphosphonate: Slows the release of calcium, blocking the mineralization of the bone matrix	3	Worsened myalgia and asthenia and onset of low-grade fever	Manifestations of POH progressed	(14)
Itraconazole	6.6 mg/kg/q.d. 9.5 mg/kg/q.d.	Antifungal: acts as a potent suppressor of the Hh signaling pathway	90 30		Biochemical markers of bone formation returned to previous levels, and absence of clinical improvement in the disease progression	(15)
Methylprednisolone	20 mg/kg/q.d. Slow tapering	Corticosteroid hormone	5 180		Absence of clinical improvement in the disease progression, despite reuction of markers of bone formation after the initial bolus.	(2, 16)
Indomethacin	3 mg/kg/b.i.d 4 mg/kg/b.i.d	NSAIDs	180		Currently on indomethacin.	(17, 18)

*AL, administration length (days); b.i.d, bis in die (twice daily); q.d, quaque die (once a day)*.

Our novel IGF-1 therapeutic approach was based on its role as an equipoise of bone metabolism (Figure 1) (6–8), it acts as a powerful regulator of osteoclastogenesis and the reabsorptive function of osteoclasts (19). Moreover, immunological experiments in animals genetically modified to nullify the expression of the gene for IGF-1 showed excessive and uncontrolled growth of bone tissue due to osteoclastic inhibition (20). After administration of IGF-1 to our patient, serum levels of substances thought to upregulate osteoclasts (Beta-crosslaps) rose by 10% (1.71 pre-intervention to 1.87 ng/ml post-intervention), whereas PINP (promoting osteoblasts) concentrations rose by 30% (from 853.6 to 1177 ng/ml). This effect is similar to the results obtained from IGF-1 administration in both healthy subjects and patients with other pathologies (21, 22).

In our case, decreased IGF-1 values seem to be the result of the underlying altered molecular pathways found in POH and not a cause or a key player. It may also be possible that subcutaneous administration of IGF-1 has no effect on bone formation or that higher doses are required to elicit a response. Our results may also be influenced by the fact that IGF-1 acts in the latter stages of bone maturation (22). Low levels of IGF-1 can also translate to an impaired GH secretion (23). Whenever tested, serum GH concentrations were found to be in adequate levels for its age in our patient, and the same happened to IGFBP-3 concentrations (a protein binding IGF-1). Although maternally derived *GNAS* mutations may lead to GHRH resistance and therefore low circulating levels of GH, most cases of POH are caused by heterozygous inactivating mutations of the *GNAS* gene on the paternal allele. What is more, alterations in paternal and maternal alleles drive to strikingly distinct phenotypes—slim in paternally—inherited mutations (as happened to our subject), and obesity in *GNAS* disturbances of maternal origin (24). We did not test the patient's response to provocative test for GH secretion; reproducibility remains an issue with these tests, and at the time of the initial assessment, the severely affected girl did not comply with the consensus criteria proposed for them (23). Our clinical and analytical work-up also suggested the absence of a concomitant hypopituitarism. Lastly, lower levels of IGF-1 can also be found in states of poor nutrition (23), and the continued bone formation of POH may have likely had an impact on metabolic expenses.

It has been suggested that retinoic acid increases the production of Gsα protein from the normal allele (10). Use of selective retinoic acid receptor agonist for inhibition of ectopic bone formation has been reported by Shimono et al. (11, 12). Isotretinoin, in fact, has been used for patients with fibrodisplasia ossificans progressiva (FOP) (13). However, retinoic acid receptor Y agonists inhibit endochondral ossification, which may be present in up to 50% patients with POH, but the typical feature of POH is the intramembranous ossification. Future studies with another retinoic (palovarotene) in FOP may shed more light on this matter (25, 26), but both courses of topical and systemic retinoids failed in stopping disease progression in our case.

Another treatment used for ectopic bone formation is thiazolidinediones, a class of antidiabetic drugs, which activate the peroxisome proliferator-activated receptor–γ (PPAR-γ) that, in bone, controls cell differentiation of mesenchymal and hematopoietic lineages. Thiazolidinedione use has been linked to increased bone resorption and decrease of bone formation (27). Thus, Gatti et al. (28) used rosiglitazone (a thiazolidinedione) during a 14-month period in a 48-year-old woman suffering from FOP, allowing a progressive tapering of the corticosteroid therapy the patient was on, and a clear improvement of joint mobility and skin softness. However, in our case, rosiglitazone was not considered due to its unknown security profile for children as young as our patient. Moreover, so far, results of an open-label study designed for further testing the effects of rosiglitazone in FOP remain undisclosed (29). It is noteworthy that this therapeutic approach is currently considered still occasionally [e.g., see the last consensus for medical management of FOP (26)].

Biphosphonates, such as Etidronate, block the mineralization of the bone matrix. In our patient, disease progression (measured by the formation of new lesions and progression of older ones) was not modified by administration of intravenous pamidronate, despite a decrease in markers of bone remodeling. Larger courses of Pamidronate are associated with “freezing” of bone metabolism, decreasing bone formation, as well as resorption (14). But for treating active flares of ectopic bone formation, evidence of such “freezing” is feeble (16, 30, 31). Etidronate has also been used for the prevention of heterotopic ossification (HO), and it seemed to retard osteoid calcification, as ossification continued when bisphosphonate was discontinued (32), because matrix formation remains unaffected (17). Despite some authors reporting pamidronate to ameliorate FOP symptoms in up to three quarters of their patients, current FOP management guidelines inform of the limited long-term utility of this approach, as repeated infusions may lead to fractures in the normal bone and biphosphonates seem to offer no protective effects against future flare-ups (26). In any case, we were not able to provide further doses of pamidronate and test the effects of a greater cumulative dosage, as pamidronate infusion was accompanied of numerous adverse events mimicking symptoms of active disease (asthenia, myalgia, pain when standing, early fatigability, etc.) lasting more than a week.

Hedgehog (Hh) signaling is required in both endochondral and intramembranous ossification (33). In normal soft tissues, Gsα inhibits Hh signaling and restricts spatially the bone formation to the normal skeleton. Hh upregulation is both necessary and sufficient to induce HO (34). At the same time, the loss of Gsα inhibition on Hh signaling can lead to the development of medulloblastoma (35). Itraconazole is an orally administered antifungal that acts as a potent suppressor of the Hh signaling pathway. The same dosage used for treating fungal infections inhibits the growth of medulloblastoma in mouse allograft models (15). To the best of our knowledge, there are no previous reports of itraconazole use in HO (26), but being itraconazole a drug frequently used in immunosuppressed children, it was ruled into the protocol. Disappointingly, after four months on itraconazole, the patient presented the formation of two new plates and the progression of older ones, and the drug was discontinued.

Corticosteroids are drugs with immunomodulatory and anti-inflammatory potential. Dosages of 2 mg/kg/once daily for four days are indicated for FOP as first-line treatment at the beginning of the flare-ups (26, 36). It is reported that up to 55% of subjects suffering from FOP experiment have some kind of symptom improvement after its use, but current protocols advise against its long-term use and restrict this treatment option to flare-ups impairing mobility of major joints, to primary prevention after severe trauma or whenever undergoing a surgery (26). We infused a high dose of corticosteroid (20 mg/kg of daily intravenous methylprednisolone, for five days) followed by a slow six-month tapering period. Although chemical parameters of bone formation dropped, laboratory findings were not accompanied by any modification of visible ectopic bone formation. A course of oral corticosteroids was previously offered by Morales et al. (16) to their patient to no benefit.

**Figure 2:**
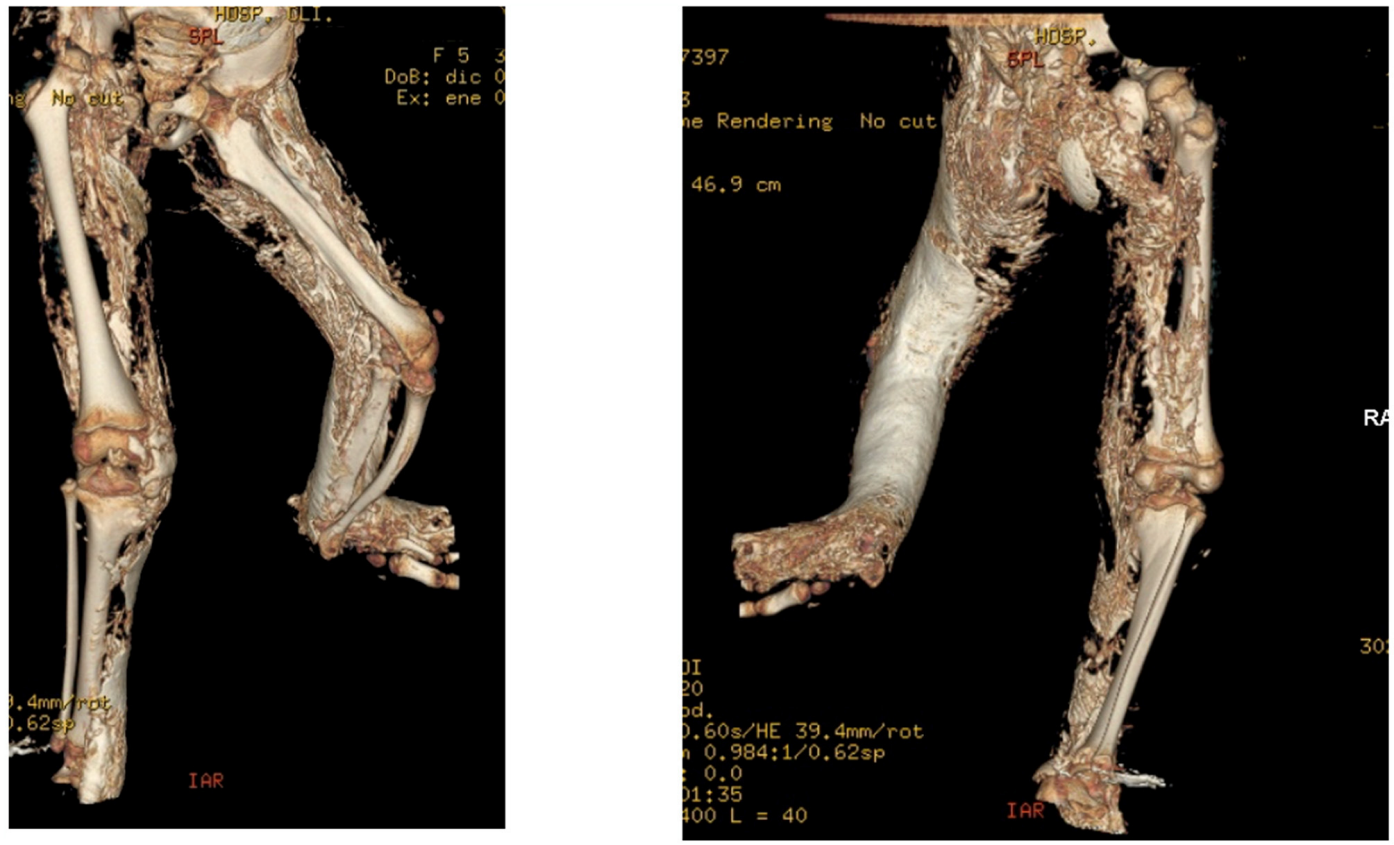
Tridimensional TC of the pelvis and lower limbs, showing the evolution of ectopic calcification in the right leg, as well as the progression of ankylosis, asymmetry, and tibial combing of the left leg.

Non-steroidal anti-inflammatory drugs (NSAIDs) like indomethacin are supposed to inhibit prostaglandin production, avoiding mesenchymal cell differentiation. Its use in the prevention of HO after hip arthroplasty (17) and spinal cord injury (SCI) (32) is well-established. On the other hand, evidence for this therapeutic approach for flare-up prevention in FOP is lacking (26). Indomethacin has been previously tried on another POH patient (18), but it did not stop worsening. Despite receiving indomethacin for the last 14 months, our patient presented two flare-up periods and recurrence of the bony plate under the surgical incision (Figure 2 and Supplementary Figure 4). Also, we lack data on PINP and beta-crosslaps during indomethacin administration, as regular blood tests were discontinued to avoid further visits.

Our report is not the first one communicating the astonishingly different clinical expression of the disease in two siblings (37) nor within one family (38), but to the best of our knowledge is the only one with POH affecting two identical twins carrying the same mutation at the GNAS gene. It was known that a specific mutation pattern within *GNAS* could not predict the severity nor progression of the disease (39–41), but until today, we have no evidence that two patients with identical genetic backgrounds could have such a different evolution.

Laboratory tests are therefore of limited utility in POH patients (16, 42–49). Markers that show an increased bone turnover have been reported by Seror et al. (30) and Hou (31). We found a huge unbalance between PINP and beta-crosslaps, but we could not correlate their values with disease activity. Through most of follow-up, levels of PINP were constantly elevated, save for the periods following administration of pamidronate and methylprednisolone. Although a blatant decrease of both parameters was seen after bisphosphonate infusion, and that drop of PINP was accompanied by a rise in bone resorption when pulses of methylprednisolone were given, in no case that change in concentration of biochemical markers was followed by a relapse of the disease. It is also worth noting that IGF-1 remains low two years after discontinuation of its ectopic administration (63 ng/ml, normal range 80–233 ng/ml).

Hypermetabolism, ankylosis of left leg, and retraction in right inferior member have taken their toll on Patient 1, who is progressively separating from her height and weight percentiles. Lesions can recur after surgical removal (16, 18, 43, 50), as happened to our patient. So far, none of our therapeutic approaches worked. This may be due to insufficient dosage, but the most likely reason is that the nature of POH ossification is different from that induced by FOP and HO, together with our unknowledge of the pathological model that drives ectopic bone formation in POH. There have been reports of other potential treatments, like radiation therapy (14, 51) and Imatinib (26, 52–54), but the effects of their use in children with disseminated ectopic bone formation would be deleterious. Using a control group for POH patients is utopic, even for our case, who had his genetic identical twin developing a total distinct phenotype but remains largely unaffected. To date, diligent skin care and physical therapies are the only recommendations we can make (47), and sharing our experience on this blatantly ignored entity may help others to overcome the lack of information on *in vivo* results.

## Conclusions

We presented a unique case of POH in two genetically identical patients with totally discordant clinical courses. They confirm that another unknown molecular mechanism beyond a *GNAS* mutation should be accountable for this wide phenotype variation. We failed at finding any medication that could ameliorate the symptoms of POH.

## Data Availability Statement

The original contributions presented in the study are included in the article/Supplementary Material, further inquiries can be directed to the corresponding author.

## Ethics Statement

The studies involving human participants were reviewed and approved by Ethical Committee of Clinical Investigation of Galicia (CEIC ref. 2019/325). Written informed consent to participate in this study was provided by the participants' legal guardian/next of kin.

## Author Contributions

AJ-G conducted the literature review, devised and implemented the protocol, and wrote the original manuscript. IR-C and JG-R implemented the protocol and edited the manuscript. MC-T, SP, MC-L, AG-C, and JP-S were implied in laboratory research and data curation and made vital contributions to the paper, including the creation and adaptation of the treatment table and flow chart. MF-S and RM-G made fundamental contributions to the original manuscript and approved the final version. AS was implied in genetic testing, collaborated in writing the original manuscript, and approved the final version of the submitted paper. FM-T was granted funding for this research, devised, and supervised the application of the protocol, collaborated in writing the original manuscript, and approved the final version of the submitted paper. All authors approved the final manuscript as submitted and agree to be accountable for all aspects of the work.

## Conflict of Interest

The authors declare that the research was conducted in the absence of any commercial or financial relationships that could be construed as a potential conflict of interest.

## Supplementary Material

Supplementary Figure 1 This AP radiographs the evident web-like pattern of the POH. This reflects the clinical situation of the patient before starting the different treatments. Note the affected growth of the left leg, ankylosis of left knee and ankle, luxation of several joints of the left lower limb and foot (knee, ankle, tarso-metatarsal, etc.), and combing, resorption, remodeling, and cortical fractures of large bones. Also, a bony plate is visible in the dermis over the right knee. Although palpable, at that time, it did not greatly impaired mobility of this joint.

Supplementary Figure 2 Heatmap of identity-by-descent (IBD) values represented as p(IBD = 2) + 0.5 × p(IBD = 1) obtained from pairwise genome profile comparisons of the samples collected from the patients (coded as P1 and P2 in the figure). The data indicate that both sisters are genetically identical and do not show detectable mosaicism in the samples analyzed. IBD values for all pairs of genetic profiles are compatible with a twin relationship or samples coming from the same biological source (55); according to the Lee test (56), this relationship is highly statistically significant (*p*-value < 10^−16^ in all pairwise comparisons). We used the Applied Biosystems™ Axiom™ Spain Biobank Array to genotype blood and saliva samples from Patient 1 and Patient 2, and a bone plate sample from the severely affected Patient 1. IBD values for all pairwise comparisons were obtained for a total of 715,195 SNPs. To infer kinship relations based on the genome evidence, we followed the procedures previously described in (57, 58). We used 10 random Spanish genome profiles obtained from The 1,000 Genomes Project (IBS-1000G) (indicated with prefix “HG” in the figure) as reference samples for IBD computation.

Supplementary Figure 3 Original flowchart of therapeutic options after IGF-1 administration for Patient 1. rhIGF-1 was the first treatment provided due to the persistent low levels detected since her birth. NSAIDs were provided for symptomatic relief whenever necessary. Our protocol also involved the extraction of blood samples previous to, during, and after stopping treatment, and, whenever a plate required removal, the storage of both calcified and surrounding mesenchymal tissue for *in vitro* treatment testing.

Supplementary Figure 4 Taken before starting corticoid therapy. It shows the progression of heterotopic bone formation in the right lower limb, especially over the medial surface of the tibia. Calcification has affected also part of the right ankle and the dermis over the adductors. The asymmetry between both lower limbs is evident (it has progressed since the initial radiograph), as well as ankylosis of both left knee and ankle. The surgical incision is the result of the removal of a bony abdominal plate that caused pain when sitting, which coupled with the impossibility of prolonged standing due to evident motives and impacted heavily on the quality of life. Shortly after taking this image, the patient underwent an orthopedic surgery for liberating the right knee, whose movements were also limited. The right leg was the one allowing walking in the patient after total ankylosis of the left leg.

## Abbreviations

FOP: Fibrodisplasia Ossificans Progressiva
Gsα: α-subunit of the G-protein
Hh: Hedgehog
HO: Heterotopic ossification
IGF-1: Insulin-like Growth Factor-1
NSAIDs: Non-Steroidal Anti-Inflammatory Drugs
PINP: procollagen type 1 N-terminal propeptide
POH-: Progressive Osseous Heteroplasia
rhIGF-1: Mecasermin (recombinant human Insulin-like Growth Factor-1)
SCI: Spinal Cord Injury.
