# Corrigendum: Case Report: Two Monochorionic Twins With a Critically Different Course of Progressive Osseous Heteroplasia

**DOI:** 10.3389/fped.2021.807812

**Published:** 2021-12-14

**Authors:** Antonio José Justicia-Grande, Jose Gómez-Ríal, Irene Rivero-Calle, Sara Pischedda, María José Curras-Tuala, Alberto Gómez-Carballa, Miriam Cebey-López, Jacobo Pardo-Seco, Roberto Méndez-Gallart, María José Fernández-Seara, Antonio Salas, Federico Martinón-Torres

**Affiliations:** ^1^Genetics, Vaccines, Infectious Diseases and Pediatrics Research Group (GENVIP Group), Instituto de Investigación Sanitaria de Santiago de Compostela, Santiago de Compostela, Spain; ^2^Physical Medicine and Rehabilitation Department, Hospital Clínico Universitario de Santiago de Compostela, A Coruña, Spain; ^3^Immunology Laboratory, Clinical Laboratory, Hospital Clínico Universitario Santiago de Compostela, A Coruña, Spain; ^4^Translational Pediatrics and Infectious Diseases, Department of Pediatrics, Hospital Clínico Universitario de Santiago de Compostela, A Coruña, Spain; ^5^Pediatric Surgery, Hospital Clínico Universitario de Santiago de Compostela, A Coruña, Spain; ^6^Unidade de Xenética, Instituto de Ciencias Forenses, Facultade de Medicina, Universidade de Santiago de Compostela, Santiago de Compostela, Spain; ^7^GenPoB Research Group, Instituto de Investigaciones Sanitarias, Hospital Clínico Universitario de Santiago de Compostela, A Coruña, Spain

**Keywords:** progressive osseous heteroplasia, POH, treatment, genetic diseases, monochorionic twins

In the original article, there was an error. “Osseous” was misspelled as “Osseus” in the title.

A correction has been made to the **Title**:

“Case Report: Two Monochorionic Twins With a Critically Different Course of Progressive Osseous Heteroplasia”

The authors apologize for this error and state that this does not change the scientific conclusions of the article in any way. The original article has been updated.

## Publisher's Note

All claims expressed in this article are solely those of the authors and do not necessarily represent those of their affiliated organizations, or those of the publisher, the editors and the reviewers. Any product that may be evaluated in this article, or claim that may be made by its manufacturer, is not guaranteed or endorsed by the publisher.

