# Supplementary figures and images for: Case Report: Two Monochorionic Twins With a Critically Different Course of Progressive Osseous Heteroplasia
Source: Front Pediatr. 2021 Jun 23;9:662669. doi: 10.3389/fped.2021.662669 (PMC8260848; doi:10.3389/fped.2021.662669)

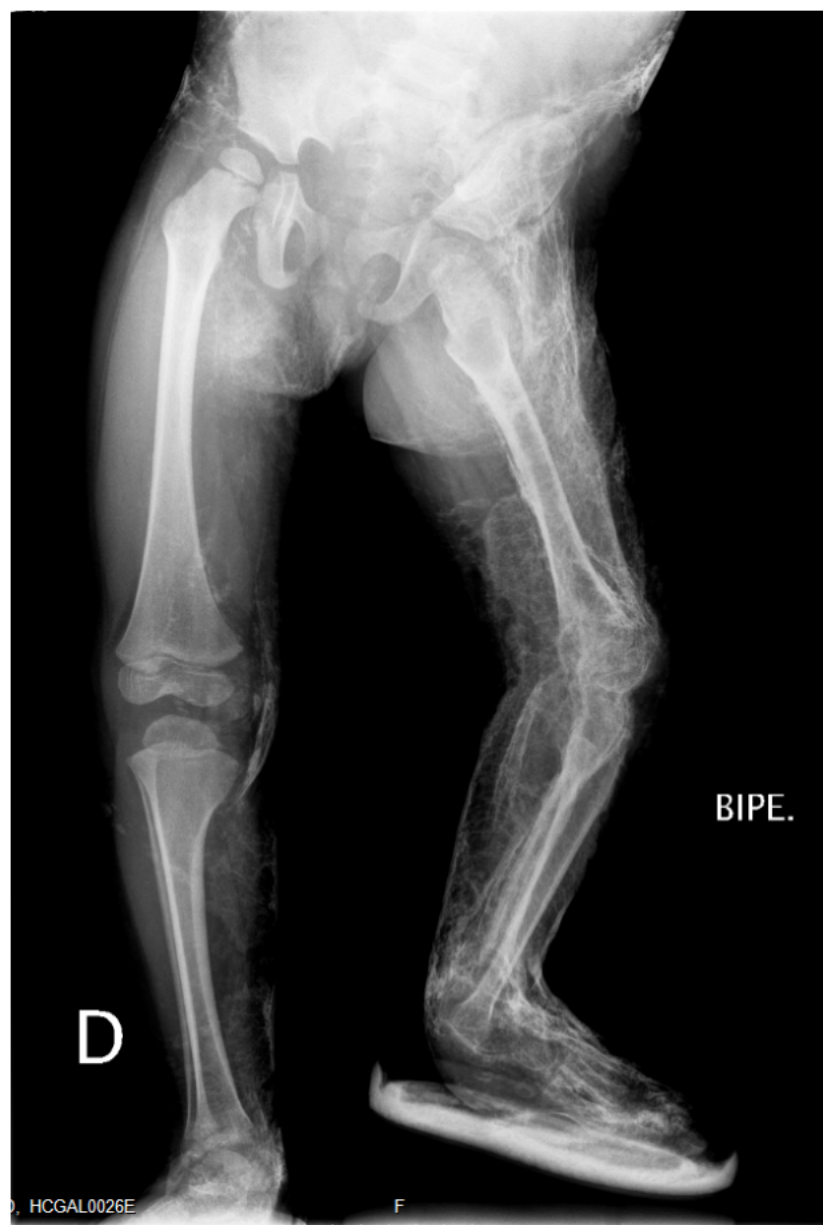

Supplement: Supplementary Figure 1 — This AP radiographs the evident web-like pattern of the POH. This reflects the clinical situation of the patient before starting the different treatments. Note the affected growth of the left leg, ankylosis of left knee and ankle, luxation of several joints of the left lower limb and foot (knee, ankle, tarso-metatarsal, etc.), and combing, resorption, remodeling, and cortical fractures of large bones. Also, a bony plate is visible in the dermis over the right knee. Although palpable, at that time, it did not greatly impaired mobility of this joint. [file Data_Sheet_1.PDF]

**NAPROXEN**  
(7.5 mg/kg/12h)

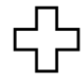

**TOPICAL RETIROID**

Scheme:

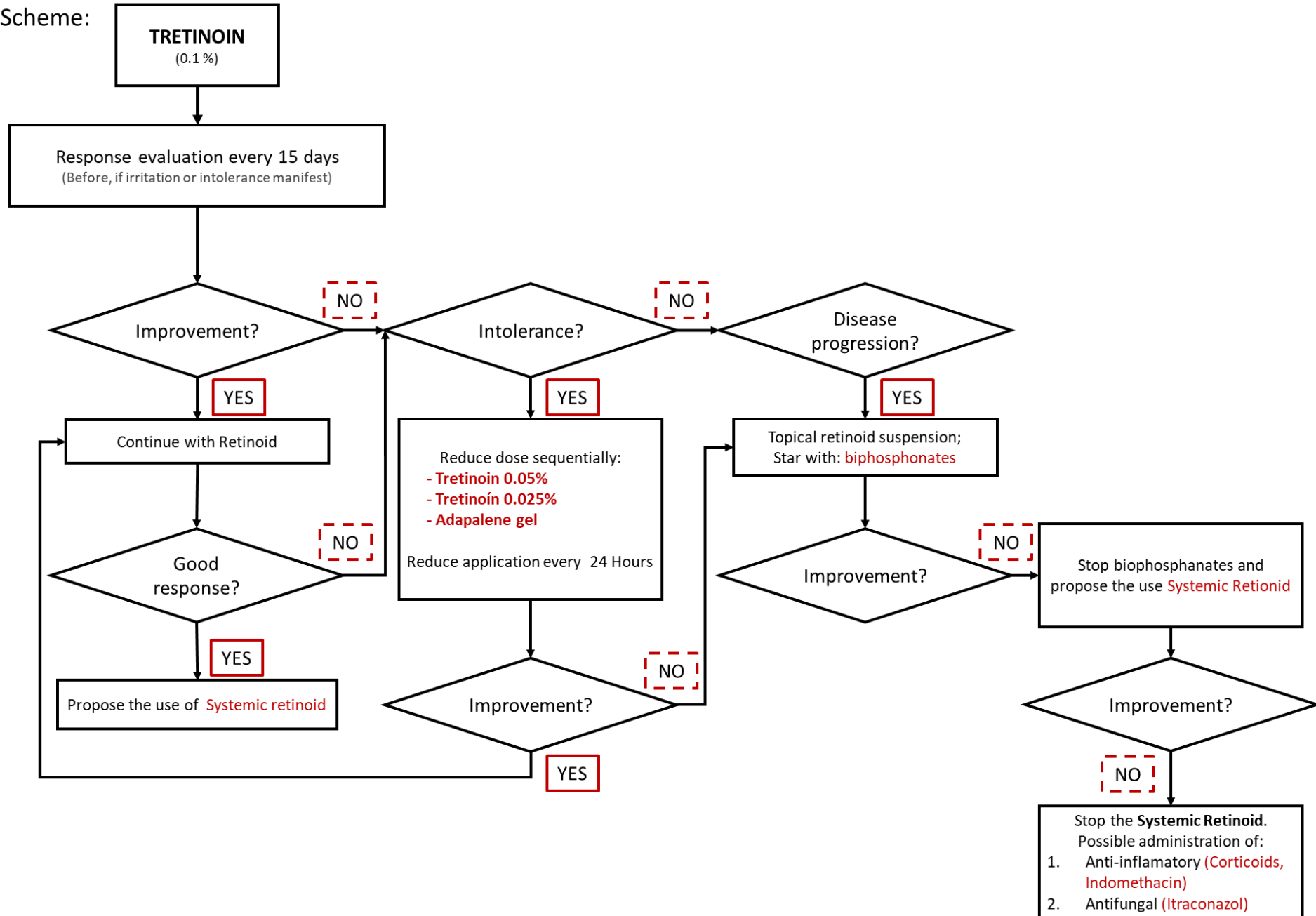

Supplement: Supplementary Figure 3 — Original flowchart of therapeutic options after IGF-1 administration for Patient 1. rhIGF-1 was the first treatment provided due to the persistent low levels detected since her birth. NSAIDs were provided for symptomatic relief whenever necessary. Our protocol also involved the extraction of blood samples previous to, during, and after stopping treatment, and, whenever a plate required removal, the storage of both calcified and surrounding mesenchymal tissue for in vitro treatment testing. [file Data_Sheet_3.PDF]

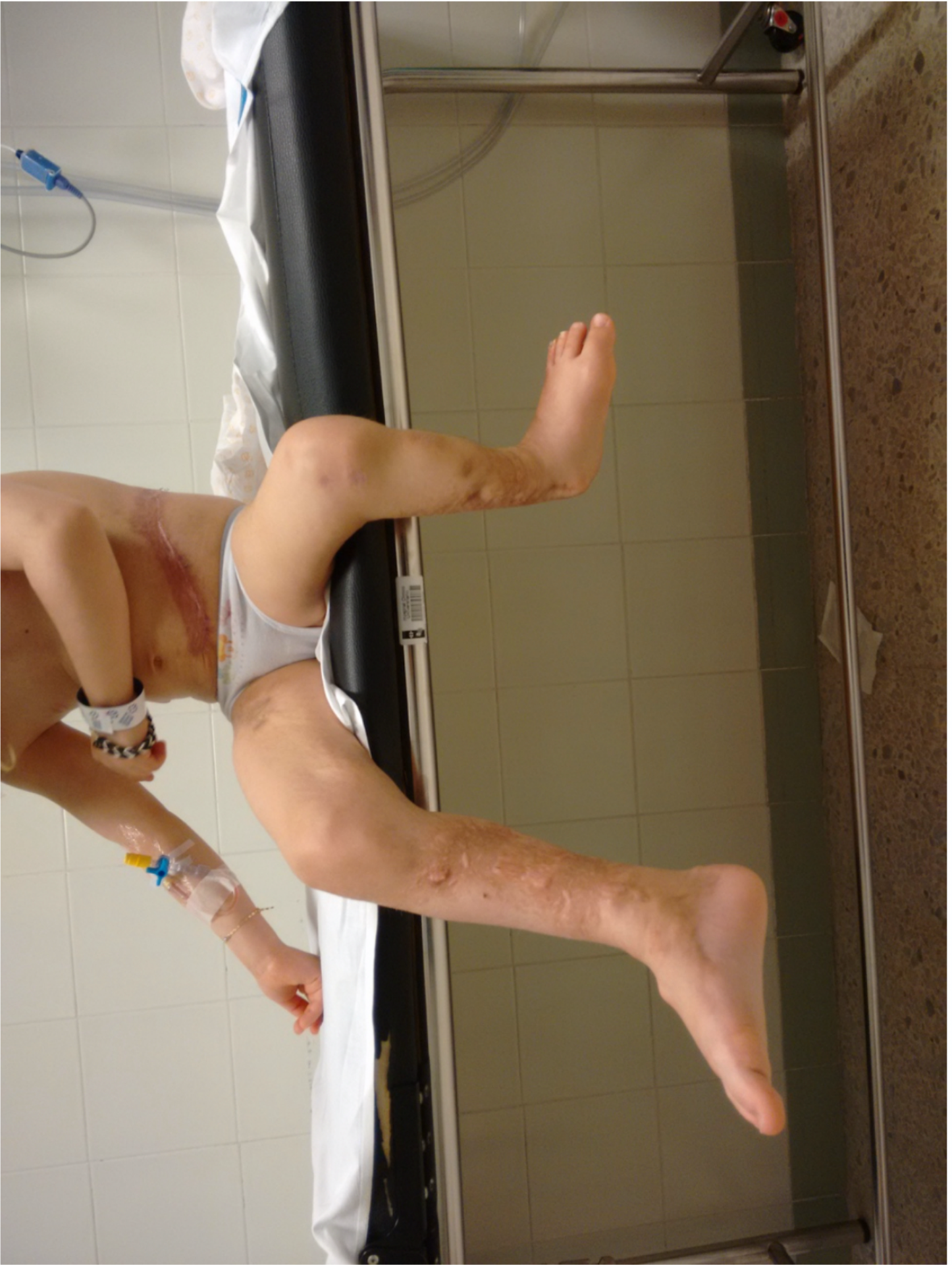

Supplement: Supplementary Figure 4 — Taken before starting corticoid therapy. It shows the progression of heterotopic bone formation in the right lower limb, especially over the medial surface of the tibia. Calcification has affected also part of the right ankle and the dermis over the adductors. The asymmetry between both lower limbs is evident (it has progressed since the initial radiograph), as well as ankylosis of both left knee and ankle. The surgical incision is the result of the removal of a bony abdominal plate that caused pain when sitting, which coupled with the impossibility of prolonged standing due to evident motives and impacted heavily on the quality of life. Shortly after taking this image, the patient underwent an orthopedic surgery for liberating the right knee, whose movements were also limited. The right leg was the one allowing walking in the patient after total ankylosis of the left leg. [file Data_Sheet_4.PDF]
