# Supplementary material for: Case Report: Two Monochorionic Twins With a Critically Different Course of Progressive Osseous Heteroplasia
Source: Front Pediatr. 2021 Jun 23;9:662669. doi: 10.3389/fped.2021.662669 (PMC8260848; doi:10.3389/fped.2021.662669)
Supplement: Supplementary Figure 2 — Heatmap of identity-by-descent (IBD) values represented as p(IBD = 2) + 0.5 × p(IBD = 1) obtained from pairwise genome profile comparisons of the samples collected from the patients (coded as P1 and P2 in the figure). The data indicate that both sisters are genetically identical and do not show detectable mosaicism in the samples analyzed. IBD values for all pairs of genetic profiles are compatible with a twin relationship or samples coming from the same biological source (55); according to the Lee test (56), this relationship is highly statistically significant (p-value < 10−16 in all pairwise comparisons). We used the Applied Biosystems™ Axiom™ Spain Biobank Array to genotype blood and saliva samples from Patient 1 and Patient 2, and a bone plate sample from the severely affected Patient 1. IBD values for all pairwise comparisons were obtained for a total of 715,195 SNPs. To infer kinship relations based on the genome evidence, we followed the procedures previously described in (57, 58). We used 10 random Spanish genome profiles obtained from The 1,000 Genomes Project (IBS-1000G) (indicated with prefix “HG” in the figure) as reference samples for IBD computation. [file Data_Sheet_2.PDF]

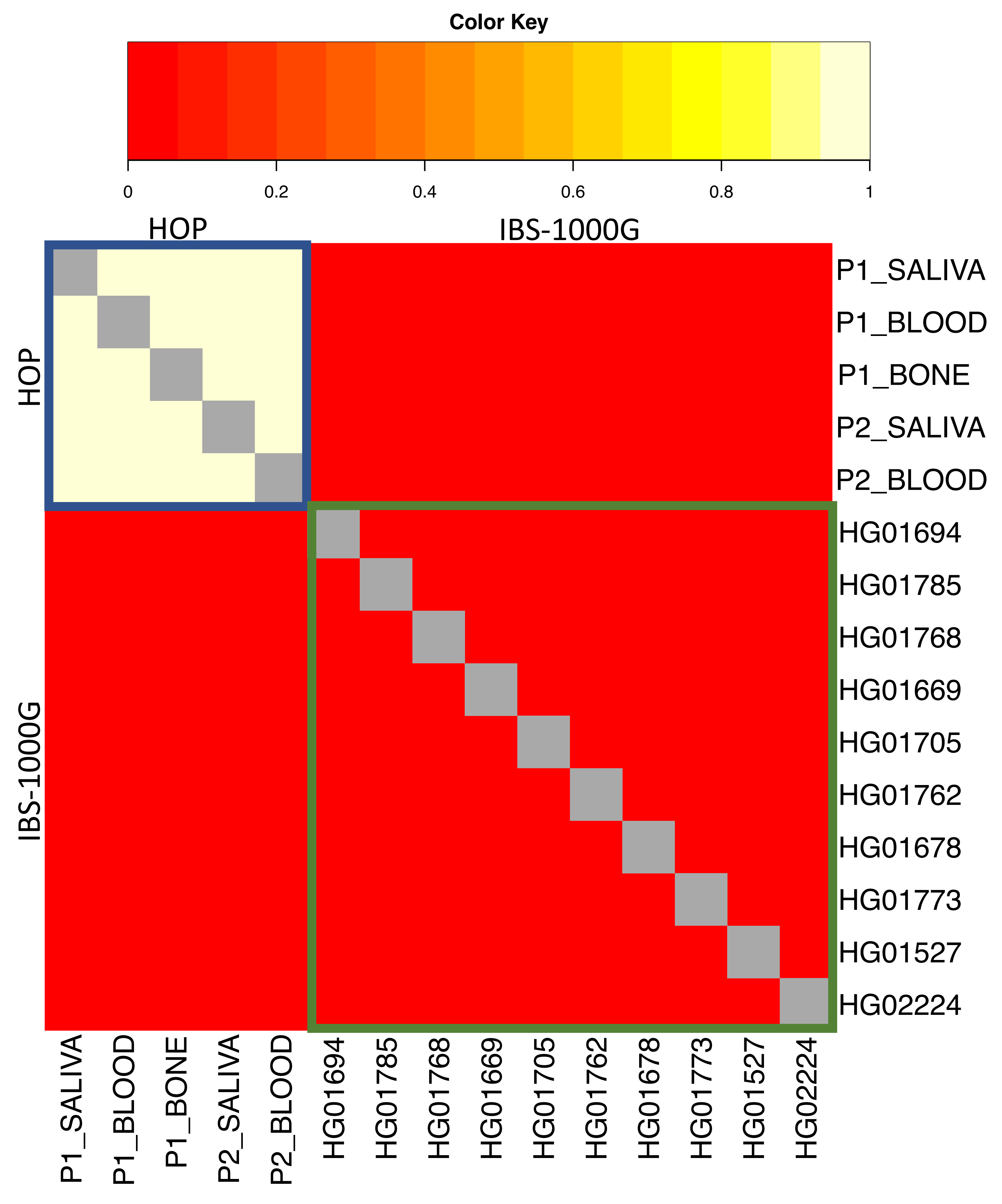
Color Key
0
0.2
0.4
0.6
0.8
1
HOP
IBS-1000G
HOP
IBS-1000G
P1_SALIVA
P1_BLOOD
P1_BONE
P2_SALIVA
P2_BLOOD
HG01694
HG01785
HG01768
HG01669
HG01705
HG01762
HG01678
HG01773
HG01527
HG02224
P1_SALIVA
P1_BLOOD
P1_BONE
P2_SALIVA
P2_BLOOD
HG01694
HG01785
HG01768
HG01669
HG01705
HG01762
HG01678
HG01773
HG01527
HG02224
